# Functional Change of Effector Tumor-Infiltrating CCR5^+^CD38^+^HLA-DR^+^CD8^+^ T Cells in Glioma Microenvironment

**DOI:** 10.3389/fimmu.2019.02395

**Published:** 2019-10-09

**Authors:** Pin-Yuan Chen, Caren Yu-Ju Wu, Jian-He Fang, Hsiu-Chi Chen, Li-Ying Feng, Chiung-Yin Huang, Kuo-Chen Wei, Jia-You Fang, Chun-Yen Lin

**Affiliations:** ^1^Department of Neurosurgery, Linkou Chang Gung Memorial Hospital, Taoyuan, Taiwan; ^2^Department of Neurosurgery, Keelung Chang Gung Memorial Hospital, Keelung, Taiwan; ^3^School of Medicine, Chang Gung University, Taoyuan, Taiwan; ^4^Pharmaceutics Laboratory, Graduate Institute of Natural Products, Chang Gung University, Taoyuan, Taiwan; ^5^Graduate Institute of Biomedical Sciences, Chang Gung University, Taoyuan, Taiwan; ^6^Department of Gastroenterology and Hepatology, Linkou Chang Gung Memorial Hospital, Taoyuan, Taiwan; ^7^Research Center for Food and Cosmetic Safety, Research Center for Chinese Herbal Medicine, Chang Gung University of Science and Technology, Taoyuan, Taiwan; ^8^Department of Anesthesiology, Linkou Chang Gung Memorial Hospital, Taoyuan, Taiwan

**Keywords:** glioma, CD8, T cell, CCL5, CCR5

## Abstract

Human glioma facilitates an impaired anti-tumor immunity response, including defects in circulation of T lymphocytes. The level of CD8^+^ T-cell activation acts as an immune regulator associated with disease progression. However, little is known about the characteristics of peripheral and tumor-infiltrating CD8^+^ T cells in patients with glioma. In this study, we examined the level of CD8^+^ T-cell activation in a group of 143 patients with glioma and determined that peripheral CD3^+^ T cells decreased in accordance with disease severity. The patients' peripheral CD8^+^ T-cell populations were similar to that of healthy donors, and a small amount of CD8^+^ tumor-infiltrating lymphocytes was identified in glioma tissues. An increase in activated CD8^+^ T cells, characterized as CD38^+^HLA-DR^+^, and their association with disease progression were identified in the patients' peripheral blood and glioma, and shown to display enriched CCR5^+^ and TNFR2^+^ expression levels. *Ex vivo* examination of CD38^+^HLA-DR^+^CD8^+^ T cells indicated that this subset of cells displayed stronger secretion of IFN-γ and IL-2 before and after a 6-h stimulation with phorbol 12-myristate 13-acetate (PMA) and ionomycin (ION) relative to healthy CD38^+^HLA-DR^+^CD8^+^ T cells, indicating the functional feasibility of CD38^+^HLA-DR^+^CD8^+^ T cells. Higher CCL5 protein and mRNA levels were identified in glioma tissues, which was consistent with the immunohistochemistry results revealing both CCL5 and CD38^+^HLA-DR^+^CD8^+^ T cell expression. Patients' CCR5^+^CD38^+^HLA-DR^+^CD8^+^ T cells were further validated and shown to display increases in CD45RA^+^CCR7^−^ and T-bet^+^ accompanied by substantial CD107-a, IFN-γ, and Granzyme B levels in response to glioma cells.

## Introduction

Gliomas are tumors that arise from glial precursor cells and include astrocytoma, oligodendroglioma, ependymoma, mixed glioma, and glioblastoma (GBM) (1), and their malignancy is graded as low grade (GI and GII) or high grade (GIII and GIV) (2). Amongst gliomas, glioblastoma multiforme (GBM; GIV) is the most lethal type of these malignant brain tumors with a median survival of 15 months despite multimodal therapies, including surgery followed by radiation and temozolomide therapy (2, 3). The recurrence of GBM usually takes place around the initial resected lesion (within 2 cm), which offsets the effects of standard therapeutic approaches on overall survival (2, 4). The immune response of patients with glioma are characterized by defects in circulating T-cell populations, poor tumor antigen-specific CD8^+^ T-cell responses, and elevated programmed death 1 (PD-1) in CD8^+^ T cells contributing to the poor prognosis of these patients (5–9). Previously, low tumor-infiltrating CD8^+^ cells (CD8^+^ TILs) were reported to associate with poor progression-free survival (PFS), defined as from the date of surgery to the first MRI-confirmed recurrence (10, 11).

The CD8^+^ cytotoxic T cells are functional/effector cytotoxic T lymphocytes (CTLs) in which only 5–10% of the original burst size matures into long-lived protective memory CD8^+^ T cells (12). In addition, autocrine IL-2 produced by CD8^+^ T cells activated via interaction with APC pre-activated by CD4^+^ T cells is important for the generation of competent CTLs (13). The presence of CD8^+^ T cells was shown to reduce tumorigenicity when CD4^+^ or NK^+^ cell-depleted mice had been immunized with IL-7-producing glioma cells (14). In addition, local application of an AC133/CD133-specific T-cell-engaging antibody (a cancer stem cell marker) with human CD8^+^ T cells has been shown to prevent tumor outgrowth of subcutaneous GBM xenografts (8). Recently, blockage of PD-1 using RMP1-14 (15) and IL-2-treated CD8^+^ cells coinciding with IL-2R abundance have also been shown to consolidate CD8^+^ T cells' functional viability (16). However, the mechanisms underlying this phenomenon along with the phenotypic presentation of the CD8^+^ TILs are not clear.

Evidence has revealed that CD38 and HLA-DR are common effector T-cell activation markers that also associate with disease states, including autoimmune diseases, HIV, leukemia, and multiple myeloma (17–19). Expression of CD38 on tumor cells was indicated to serve as a negative prognostic marker implying disease severity and poor survival (20). The functional expression of CD38 and HLA-DR was reported to reflect infection status as it related to survival capacity in glioma (18, 19, 21, 22). Furthermore, Hua et al. (18) indicated that the increased expression of CD38^−^HLA-DR^+^CD8^+^ HIV-specific T cells displayed a better survival capacity than the expression of CD38^+^HLA-DR^+^CD8^+^ cells. In addition, the functional expression of C-C chemokine receptor 5 (CCR5) was demonstrated to be critical for effector CD8^+^ T-cell migration to inflamed tissues, in which the production of INF-α, IL-2, and IFN-γ were positive for CCR5 potentiation (23, 24). The association of increased CCR5 expression with viral susceptibility and viral entry was evidenced in HIV and cytomegalovirus (CMV) studies (25, 26). As such, the current study sought to identify the basic immune functional capacity among patients with glioma for clinical reference. In addition, the role of the effector CD8^+^ T cells in patients with glioma remains unclear. Therefore, we are interested in knowing whether the effector CD8 T^+^ cells are contributable toward glioma development and their role in tumor regression.

In the present study, we investigated the activation of the CD38^+^HLA-DR^+^CD8^+^ subpopulation among total human CD8^+^ cells along with their penetration into the tumor microenvironment in a large series of glioma samples. We demonstrated an association between increases of glioma CCL5 and CCR5^+^CD38^+^HLA-DR^+^CD8^+^ TILs. In the presence of glioma cells, this CD8^+^ T cell subset appears to facilitate the enhancement of effector molecules, including CD45RA^+^CCR7^−^, T-bet^+^, CD107a^+^, IFN-γ^+^, and granzyme B^+^.

## Materials and Methods

### Standard Protocol Approval, Registration, and Patient Consent

This study was approved by the Chang Gung Medical Foundation Institutional Review Board (No. 102-1096B) and in accordance with the Helsinki Declaration. Patients were recruited from the Department of Neurosurgery, Chang Gung Medical Foundation, Taiwan, between 2013 and 2016. All eligible patients were informed about the details of this study and provided a signed informed consent prior to study participation.

### Glioma Cell Culture

Human U87 glioblastoma cells were obtained from the American Type Culture Collection (ATCC, Manassas, VA). Cells were maintained in MEM supplemented with 10% FBS, 100 U/ml penicillin, and 100 mg/mL streptomycin. Cells were incubated at 37°C in a humidified incubator with an atmosphere of 5% CO_2_ and 95% air.

### Preparation of Blood and Tumor Samples

Blood and freshly resected tumor materials were collected from the participants with primary glioma, who had been diagnosed histopathologically according to the current WHO criteria. Peripheral blood mononuclear cells (PBMCs) from healthy donors and patients with glioma were isolated by Ficoll density gradient centrifugation (Ficoll-Paque; GE Healthcare Life Sciences, Sweden) as described by Lefort and Kim (27). Tumor material was obtained from patients who had received surgical excision. Tissues were collected into medium (F12; Gibco, USA) containing 10% FBS and 5% P/S, and these were processed under sterile conditions as previously described (28). Briefly, tumor tissues were washed several times with PBS to remove the majority of erythrocytes. Tissues were cut into 1–2 mm pieces, digested with 0.05% trypsin for 40 min at 37°C, extensively filtered using a 70 μm strainer, and then separated on Ficoll gradients to yield single cell-enriched preparations from tumor lysates, and characterized by staining of CD3, CD8, CD38, HLA-DR, CCR5, and TNFR2 antibodies. CD8^+^ T lymphocytes were negatively selected using a CD8 T-cell isolation kit (Miltenyi Biotec, USA) that non-CD8^+^ cells including CD4^+^, CD15^+^, CD16^+^, CD19^+^, CD34^+^, CD36^+^, CD56^+^, CD123^+^, TCR γ/δ^+^, and CD235a^+^ cells are magnetically labeled, and depleted using magnetic separator. A purity of 80 ± 2% was confirmed by flow cytometry analysis with anti-CD3-FITC and anti-CD8-PerCP antibodies for CD8^+^ T lymphocytes, and there was an approximate 20% of cell debris (data not shown).

### Multiparameter Flow Cytometry Analysis

Four-color staining of lymphocytes was performed to determine the frequency and phenotype of lymphocytes among freshly isolated PBMCs from either healthy donors or patients as well as among TILs from patients. The following monoclonal antibodies (mAbs) were used: CD19-PerCP (#302228), CD56-APC (#555518), CD3-FITC (#555332), CD8-PerCP (#340693), CD8-FITC (#555366), CD45RA-FITC (#555488), CD38-FITC (#555459), CD38-APC (#555462), HLA-DR-APC (#559866), HLA-DR-BV421 (#307636), CCR5-FITC (#555992), CCR5-PE (#555993), TNFR2-PE (#FAB226P), IFN-γ-PE (#559326), IL-2-PE (#559334), PD-1-PE (#129969), Tim-3-PE (#123109), CCR7-PE-Cy7 (#557648), T-bet-PerCP-Cy5.5 (#561316), CD107a-PE (#555801), and Granzyme B-PE (#561142) (BD Biosciences, USA). Lymphocytes were first stained at 4°C for 1 h with mAbs to cell surface markers. Each sample contained at least 2 × 10^6^ lymphocytes isolated from PBMCs or TILs. Expression of surface antigens was analyzed using a dual-laser fluorescence-activated cell sorting cytofluorimeter (FACSCalibur; FACSCanto^TM^II; BD Biosciences, USA) employing Cell-Quest software (BD Biosciences, USA).

### Intracellular Cytokine Production Assays

For intracellular IFN-γ and IL-2 detection, lymphocyte and TIL suspensions were stimulated with phorbol 12-myristate 13-acetate (PMA; 50 ng/ml; Sigma-Aldrich, USA) plus ionomycin (ION; 1 μg/ml; Sigma-Aldrich, USA) for 6 h. Cells were then stained with T-cell markers, further processed using a Fixation/Permeabilization kit (BD Bioscience, USA), and then labeled with a PE-conjugated IFN-γ or IL-2 mAb (BD Biosciences, USA).

### Immunohistocytology

Triple immunohistochemistry staining was used to assess the distribution of activated cytotoxic CD8 T cells. Briefly, brain sections were prepared for immunostaining via xylene treatment and gradual rehydration with 99–75% ethanol. Sections were blocked with 1% FBS for immunohistofluorescence staining or 3% hydrogen peroxide for immunohistochemistry and then incubated with anti-CD8 (#ab17147; Abcam, USA), anti-CD38 (#ab108403; Abcam, USA), anti-HLA-DR (#17221-1-AP; Proteintech, USA), anti-PD-L1 (#13684; Cell Signaling, USA), or anti-CCL5 (#SC-1410; Santa Cruz, USA) overnight at 4°C in blocking solution. The sections were then incubated with fluorescent secondary antibodies or peroxidase-conjugated secondary antibodies for 1 h at room temperature and subsequently stained using 3,3' diaminobenzidine and counterstained with hematoxylin. Slides were coverslipped using mounting medium (Histokitt, Germany). Images were captured with a 40x objective on a Nikon Eclipse E400 microscope using SPOT software (Nikon, Japan).

### Quantitative Real-Time PCR

Total RNA was extracted from both tumor and non-tumor biopsies using TRIzol reagent (Invitrogen, USA). First-strand cDNA was synthesized from 2 μg total RNA with oligo(dT)_20_ primers and reverse transcribed to cDNA using SuperScript III First-Strand Synthesis SuperMix (Invitrogen, USA). Real-time PCR was carried out using SYBR Green I Master Mix (Roche, USA). Amplification and detection of mRNA were performed using a LightCycler 480II system (Roche, USA) under the following conditions: 40 cycles at 95°C for 10 s and 60°C for 1 m. The threshold was set within the linear phase of the target gene amplification to calculate the cycle number at which point the transcript was detected as CT. The oligonucleotide primers used for real-time PCR were:

GAPDH: 5′-CTCAACTACATGGTCTACATGTTCCA-3′and 5′-CTTCCCATTCTCAGCCTTGACT-3′;

CCL5: 5′- GAGTATTTCTACACCAGTGGCAAG-3 and 5′-TCCCGAACCCATTTCTTCTCT-3′.

### Western Blotting

Glioma cells were co-cultured with CD8^+^ T cells for 24 h using a transwell system, washed with PBS, and then lysed for 30 min on ice with RIPA lysis buffer (Thermo Fisher, MA). Protein samples were then separated by SDS-PAGEs and transferred to PVDF membranes. The membranes were blocked with 5% non-fat milk and then probed with primary antibodies against PD-L1 (Cell Signaling, MA) at 4°C overnight. After extensive washing, the membranes were incubated with secondary antibodies for 1 h at room temperature, and the blots were then visualized using Immobilon Western Chemiluminescent HRP Substrate (Millipore, MA) and Amersham Hyperfilm ECL (GE Healthcare, UK).

### Statistical Analysis

All results are expressed as means ± SE of at least three independent experiments unless stated otherwise. Due to biosample availability, not every biosample contributed to every analysis. Statistical analyses were performed using a two-tailed Student's *t*-test to determine the statistical significance between the groups using SPSS Version 19 (IBM, USA). A *p* < 0.05 was considered significant.

## Results

### Patients With Glioma Display a Decrement of Peripheral CD3^+^ T Cells in Comparison to Healthy Donors

**Figure 1:**
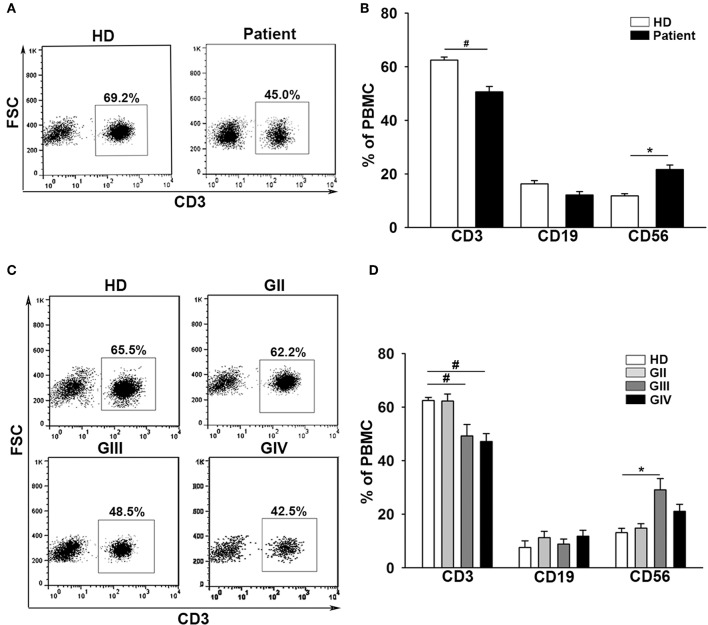
Patients with glioma display a stepwise immune deficiency in accordance with the grade classification. **(A)** PBMCs from 31 healthy donors (HD) and 117 patients (Patient) were evaluated by flow cytometry for CD3, CD19, and CD56 after gating lymphocytes. **(B)** The percentages of CD3^+^, CD19^+^, and CD56^+^ cells were quantified. **(C)** The expression of CD3^+^ decreased in accordance with the grade. **(D)** Comparisons of the percentages of CD3^+^, CD19^+^, and CD56^+^ cells across the grades are shown. Values shown are means ± SEM (bars); *p < 0.05, ^#^*p* < 0.01 by Student's *t-*test.

The baseline data for the 143 patients with glioma (mean age = 52 ± 14) and 36 healthy donors (mean age = 47 ± 16) are shown in Table 1. Patients were further characterized according to grade II (GII; *n* = 29), grade III (GIII; *n* = 30), or grade IV (GIV; *n* = 84). The independent sample *t*-test revealed no significant age difference between healthy donors (HD) and patients with glioma (*t* = −1.9, *p* = 0.06; Supplementary Tables 1, 2). Previously, immunological impairments were addressed in patients with malignant glioma with signature T-cell reduction (29, 30). We examined the proportion of the major components of lymphocytes among our patients and healthy donors using flow cytometry. Three-color staining of the surface antigens CD3, CD19, and CD56 against the lymphocyte population, representing T cells, B cells, and NK (natural killer) cells, was performed. The results revealed that the CD3^+^ population was significantly lower in patient PBMCs (Figures 1A,B) compared to healthy donors (50.6 ± 2% and 62.5 ± 1%, respectively), especially in the high grade patient group (GII = 62.3 ± 2%, GIII = 49.2 ± 4%, and GIV = 47.0 ± 3%; Figures 1C,D). On the other hand, the CD19^+^ population in PBMCs was not significantly different between the patients and healthy donors, whereas the CD56^+^ population was slightly higher in the patient PBMCs, especially in the GIII patient group (Figure 1D).

**Table 1:** Study population of healthy donors (*n* = 36) and patients with glioma (*n* = 143).

**Patient**	**Newly**	**Recurrent**	**Healthy Donor**
*N*. (male/female)	72 (43/29)	71 (46/25)	36 (18/18)
Mean age (years)	50.3 ± 14	51.3 ± 12	46.2 ± 16
Grade II (n.)	17	12	–
Grade III (n.)	16	14	–
Grade IV (n.)	39	45	–

### Increased CD38^+^HLA-DR^+^CD8^+^ T Cells and Accumulation in the Tumor Microenvironment in High-Grade Gliomas

**Figure 2:**
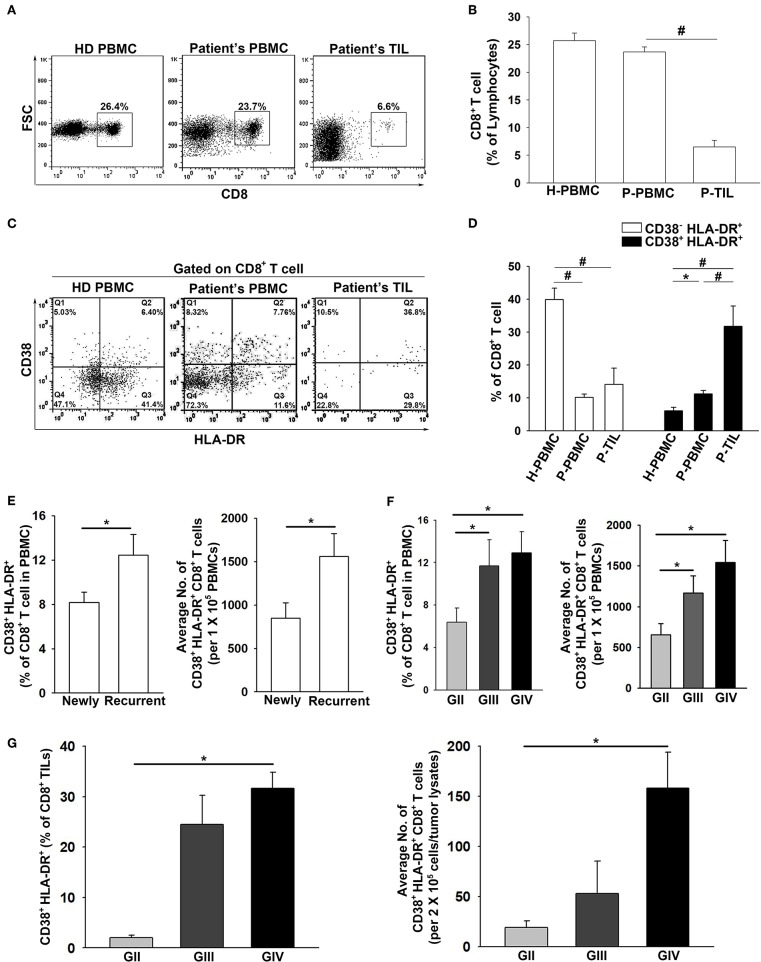
Pronounced activation and penetration of CD38^+^HLA-DR^+^CD8^+^ T cells in high-grade glioma. **(A,B)** The overall expression percentage of CD8^+^ T cells was significantly lower in Patient TILs (*n* = 17) than in Patient PBMCs (*n* = 117) and healthy donors (HD PBMC; *n* = 31). **(C,D)** The expression percentage of CD38^−^HLA-DR^+^CD8^+^ cells was higher in HD PBMCs (H-PBMCs) than in Patient PBMCs (P-PBMCs) and TILs (P-TILs); profound CD38^+^HLA-DR^+^CD8^+^ T-cell activation was observed in P-PBMCs and P-TILs. **(E)** The expression of CD38^+^HLA-DR^+^CD8^+^ T cells in newly-diagnosed (*n* = 59) and recurrent (*n* = 58) patients. **(F)** The expression of CD38^+^HLA-DR^+^CD8^+^ T cells in the PBMCs of GII (*n* = 22), GIII (*n* = 17), and GIV (*n* = 51). **(G)** The expression of CD38^+^HLA-DR^+^CD8^+^ TILs in the gliomas of GII (*n* = 3), GIII (*n* = 5), and GIV (*n* = 9). Values shown are means ± SEM (bars); *p < 0.05, ^#^*p* < 0.01 by Student's *t-*test.

The frequency of total CD8^+^ cells in the patient PBMCs and TILs were evaluated. The results showed that the CD8^+^ T population in the patient PBMCs was similar to that of the healthy donors (23.8 ± 1% and 25.7 ± 1%, respectively), whereas the presence of CD8^+^ TILs (6.5 ± 1%) was significantly lower in the glioma microenvironment compared to HD PBMCs (Figures 2A,B). In regard to the CD38^−^HLA-DR^+^CD8^+^ and CD38^+^HLA-DR^+^CD8^+^ T cells, the percentage of CD38^−^HLA-DR^+^CD8^+^ T cells was lower in both the patient PBMCs and TILs compared to that in the PBMCs from healthy donors (7.8 ± 1%, 14.1 ± 5%, and 42.5 ± 3%, respectively; Figures 2C,D). In contrast, the percentage of CD38^+^HLA-DR^+^CD8^+^ cells was higher in the patient PBMCs and more prominent in the patient TILs than in the PBMCs from healthy donors (11.2 ± 1%, 31.8 ± 6%, and 6.1 ± 1%; Figures 2C,D). Importantly, the percentage of CD38^+^HLA-DR^+^CD8^+^ cells was significantly increased in patients with recurrent glioma (newly = 8.2 ± 1%; recurrent = 12.5 ± 2%; Figure 2E); high-grade glioma (GIII and/or GIV), both in the PBMCs (GII = 7.6 ± 2%, GIII = 10.1 ± 2%, and GIV = 12.9 ± 2%; Figure 2F) and TILs (GII = 2.0 ± 1%, GIII = 24.5 ± 4%, and GIV = 31.6 ± 2%; Figure 2G).

### The Concurrent Expression of CCR5 and TNFR2 on CD38^+^HLA-DR^+^CD8^+^ T Cells From Patient PBMCs and Tumors

**Figure 3:**
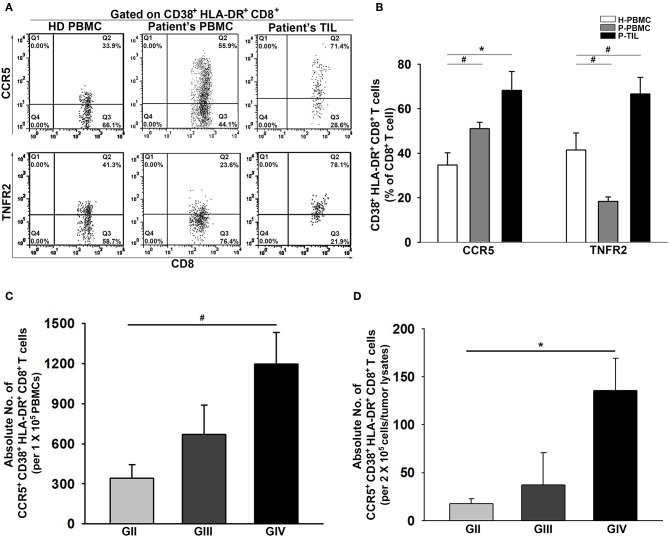
Increased activation of CCR5 and TNFR2 on CD38^+^HLA-DR^+^CD8^+^ TILs. **(A)** Representative images of percent expression of CCR5 and TNFR2 on CD38^+^HLA-DR^+^CD8^+^ T cells for HD PBMCs (*n* = 31), Patient PBMCs (*n* = 117), and Patient TILs (*n* = 17). **(B)** The percentage expression of CCR5 and TNFR2 on CD38^+^HLA-DR^+^CD8^+^ T cells were quantified. CCR5 and TNFR2 activation were inversely displayed on the patients' peripheral CD38^+^HLA-DR^+^CD8^+^ T cells; increased activation of CCR5 and TNFR2 on CD38^+^HLA-DR^+^CD8^+^ TILs. **(C,D)** Numbers of activated CCR5^+^CD38^+^HLA-DR^+^CD8^+^ T cells in the PBMCs and the gliomas of GII, GIII, and GIV were quantified. Values shown are means ± SEM (bars); *p < 0.05, ^#^*p* < 0.01 by Student's *t-*test.

The presentation of CCR5 and TNFR2 on CD38^+^HLA-DR^+^CD8^+^ T cells was further examined. As expected, the CD38^+^HLA-DR^+^CD8^+^ T cells from the patient PBMCs and tumors displayed significant upregulation of CCR5 compared to the healthy controls (34.7 ± 5%, 51.0 ± 3%, and 68.2 ± 8%; Figures 3A,B). Interestingly, a remarkable reduction in the percentage of TNFR2 expression on CD38^+^HLA-DR^+^CD8^+^ T cells in the patient PBMCs was observed, but this expression was increased in tumors. Further analysis on activated CCR5^+^CD38^+^HLA-DR^+^CD8^+^ T cells revealed a grading increment on the average numbers of CCR5^+^CD38^+^HLA-DR^+^CD8^+^ T cells presented in both patient PBMCs and tumors (Figures 3C,D).

### CD38^+^HLA-DR^+^CD8^+^ T Cells From Patient PBMCs Produce IFN-γ and IL-2 Upon Stimulation With PMA and ION

**Figure 4:**
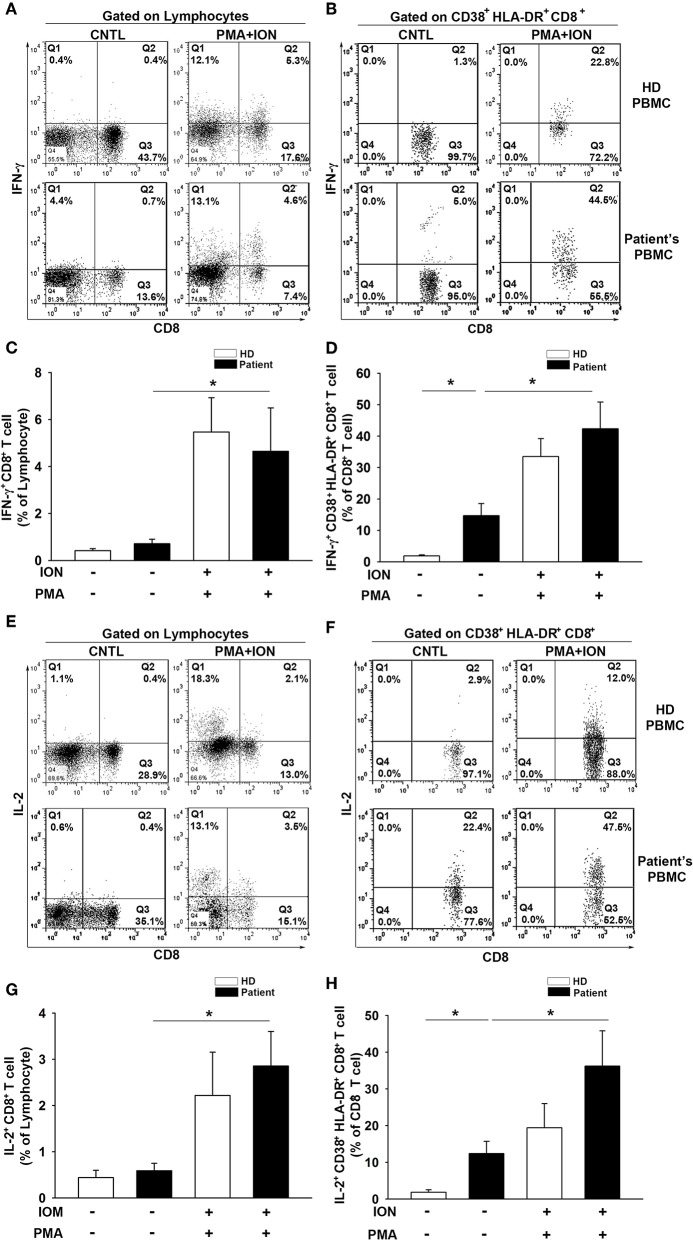
IFN-γ and IL-2 production by CD38^+^HLA-DR^+^CD8^+^ T cells from patients with glioma is enhanced. **(A)** Lymphocytes were isolated from patient (*n* = 11) and healthy donor (*n* = 10) PBMCs and then cultured for 6 h with ION (1 μg/ml) plus PMA (50 μg/ml). Intracellular cytokine production in CD8^+^ and CD38^+^HLA-DR^+^CD8^+^ T cells was analyzed. Representative images of IFN-γ production by CD8^+^ T cells before and after ION/PMA stimulation. **(B)** Representative images of IFN-γ production by CD38^+^HLA-DR^+^CD8^+^ T cells. **(C)** The percentage of IFN-γ production was increased in patient CD8^+^ T cells after ION/PMA stimulation. **(D)** The production percentage of IFN-γ by CD38^+^HLA-DR^+^CD8^+^ T cells was compared and analyzed before and after ION/PMA stimulation. **(E)** Representative images of intracellular IL-2 production by CD8^+^ T cells before and after ION/PMA stimulation. **(F)** Representative images of intracellular IL-2 production by CD38^+^HLA-DR^+^CD8^+^ T cells. **(G)** The percentage of IL-2 production by CD8^+^ T cells after ION/PMA stimulation. **(H)** Baseline IL-2 production by CD38^+^HLA-DR^+^CD8^+^ T cells was analyzed and compared after ION/PMA stimulation. CNTL = PBMCs without ION/PMA stimulation. Healthy donor = 10; patient = 11. Values shown are means ± SEM (bars); *p < 0.05 by Student's *t-*test.

To further evaluate the T-cell activation capacity, we examined the intracellular production of IFN-γ and IL-2 in CD8^+^ T cells and CD38^+^HLA-DR^+^CD8^+^ subsets from PBMCs. Cells were stimulated for 6 h with PMA (50 ng/ml) and ION (1 μg/ml) prior to analysis. Increases in both IFN-γ (HD = 0.4 to 5.5%, *t* = −3.4, *p* < 0.01; Patient = 0.7 to 4.6%, *t* = −2.1, *p* < 0.05; Figures 4A,C) and IL-2 (HD = 0.4 to 2.2%, *t* = −2.0, *p* = 0.06; Patient = 0.6 to 2.9%, *t* = −3.0, *p* < 0.01; Figures 4E,G) production were observed in total CD8^+^ T cells after PMA/ION treatment, which does not elicit a significant between-group difference for IFN-γ (HD = 5.46; Patient = 4.64, *t* = 0.34, *p* = 0.73) or IL-2 (HD = 2.21; Patient = 2.85, *t* = −0.54, *p* = 0.59) production. The baseline production of IFN-γ (HD = 0.42; Patient = 0.71, *t* = −1.34, *p* = 0.19) and IL-2 (HD = 0.44; Patient = 0.59, *t* = −0.64, *p* = 0.53) in CD8^+^ T cells was similar between the patients and healthy donors. Importantly, the baseline IFN-γ (HD = 1.88; Patient = 12.71, *t* = −3.17, *p* < 0.01; Figures 4B,D) and IL-2 (HD = 1.84; Patient = 12.41, *t* = −2.99, *p* < 0.01; Figures 4F,H) production in patient CD38^+^HLA-DR^+^CD8^+^ T cells was higher than in the healthy controls. The PMA/ION stimulation appeared to elicit a strong IFN-γ (Patient = 14.7 to 42.3%, *t* = −3.0, *p* < 0.01; Figures 4B,D) and IL-2 (Patient = 12.4 to 36.2%, *t* = −2.34, *p* < 0.05; Figures 4F,H) production in patient CD38^+^HLA-DR^+^CD8^+^ T cells. There was no significant difference in IFN-γ (HD = 33.5; Patient = 42.3, *t* = −0.85, *p* < 0.41) or IL-2 (HD = 19.4; Patient = 36.2, *t* = −1.42, *p* < 0.17) production between healthy and patient CD38^+^HLA-DR^+^CD8^+^ T cells after PMA/ION treatment. These results indicated that CD38^+^HLA-DR^+^CD8^+^ T cells from the peripheral blood of patients were already activated as effector T cells and had the capacity to receive further induction similar to the rest of the CD8^+^ cell population.

### CCL5 and CD38^+^HLA-DR^+^CD8^+^ T-Cell Presentation in Patient Tumor Tissues

**Figure 5:**
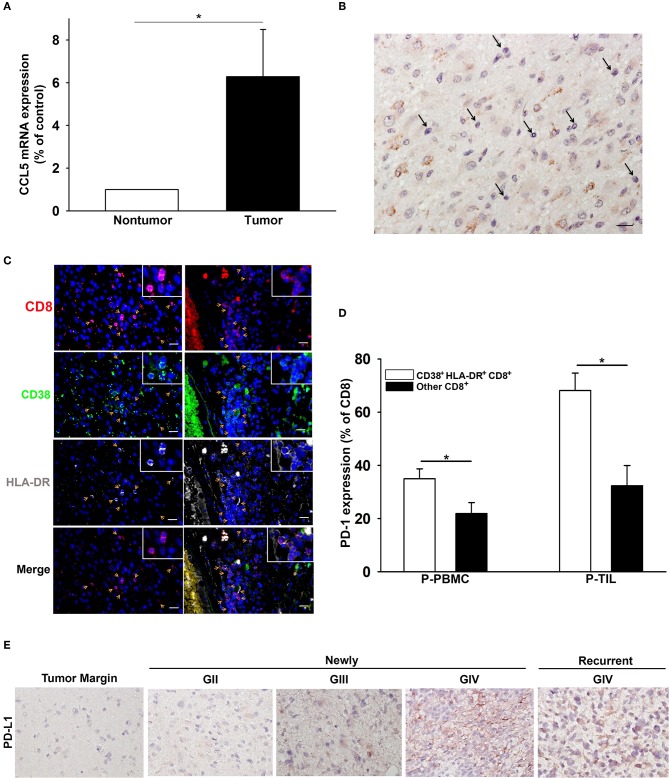
The association of CD38^+^HLA-DR^+^CD8^+^ T cell penetration and CCL5 expression in GBM. **(A)** Increased expression of CCL5 in gliomas (tumor; *n* = 15) relative to peripheral normal brain tissues (nontumor; *n* = 15). **(B)** A representative image of CCL5 (brown) expression in GBM where TILs are abundantly expressed (black arrow). **(C)** IHC revealed the co-expression (yellow arrow) of CD8 (red), CD38 (green), and HLA-DR (gray) in GBM (left column) and adjacent to blood vessels (right column). **(D)** The percentage expression of PD-1 on CD38^+^HLA-DR^+^CD8^+^ T cells (white) and other CD8^+^ T cells (black). **(E)** IHC revealed the expression of PD-L1 (brown) in GII-GIV newly-diagnosed and GIV recurrent gliomas. P-PBMCs = 38; P-TILs = 17. Scale bars = 100 μm. Values shown are means ± SEM (bars); *p < 0.05 by Student's *t-*test.

The expression of CCL5, the ligand of CCR5, was significantly higher in the glioma microenvironment than in normal peripheral brain tissues, as shown in Figure 5A. Immunohistochemical staining of glioma samples from patients with GBM revealed that TILs were located in areas where CCL5 was abundantly expressed (Figure 5B). The presence of CD38^+^HLA-DR^+^CD8 T cells was also detected by triple fluorescent staining among these TILs in GBM and areas adjacent to blood vessels (Figure 5C). Increased PD-1 expression was observed more often in CD38^+^HLA-DR^+^CD8 T cells than in the rest of the other CD8^+^ T cells obtained from the patient PBMCs and TILs (Figure 5D). We further validated the presence of CD8^+^ T cells relative to glioma PD-L1 expression. To this end, CD8^+^ T cells were isolated from PBMC of patients with newly-diagnosed and recurrent glioma, and these cells appeared to reinforce PD-L1 protein expression after co-culture with glioma cells (Supplementary Figure 1). IHC results revealed PD-L1 expression in both newly-diagnosed (GII to GIV) and recurrent gliomas (GIV) in comparison with the tumor margins, which were shown to be PD-L1 negative (Figure 5E).

### Co-expression of Functional and Exhausted Molecules in CCR5^+^CD38^+^HLA-DR^+^CD8^+^ T Cells in Response to Glioma

**Figure 6:**
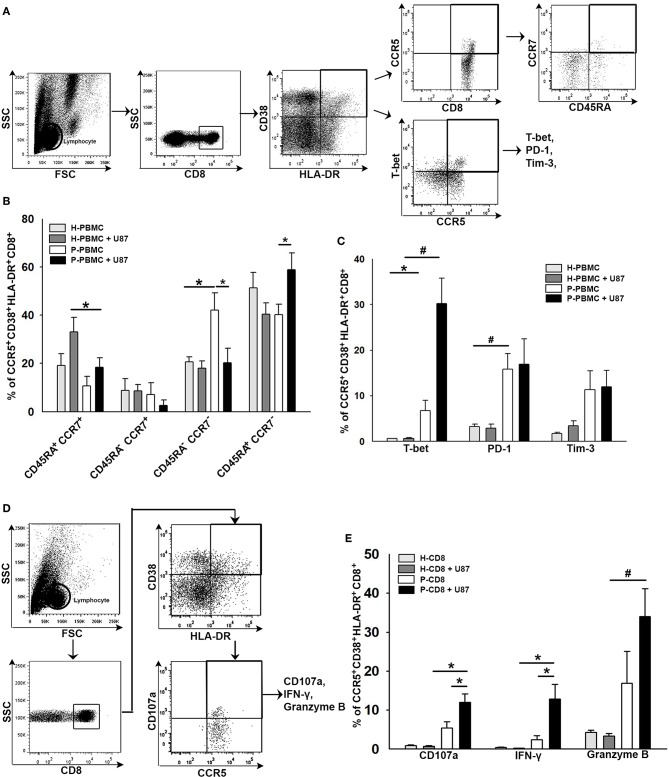
Co-expression of functional and exhausted molecules in CCR5^+^CD38^+^HLA-DR^+^CD8^+^ T cells in response to glioma. **(A)** Representative images of gating CD45RA^+^CCR7^−^ in CCR5^+^CD38^+^HLA-DR^+^CD8^+^ T cells after 24 h of co-culture with U87 glioma cells. **(B)** Responsiveness of CCR5^+^CD38^+^HLA-DR^+^CD8^+^ T cells as revealed by memory phenotypes in patients (*n* = 11) relative to HD (*n* = 6). **(C)** Expressions of T-bet, PD-1, and Tim-3 in CCR5^+^CD38^+^HLA-DR^+^CD8^+^ T cells in response to glioma (*n* = 16). **(D)** Representative images of gating CD107a, IFN-γ, and Granzyme B in CCR5^+^CD38^+^HLA-DR^+^CD8^+^ T cells after 24 h of co-culture with U87 glioma cells. **(E)** Expression of CD107a, IFN-γ, and Granzyme B in CCR5^+^CD38^+^HLA-DR^+^CD8^+^ T cells in response to glioma (*n* = 24). Values shown are means ± SEM (bars); *p < 0.05, ^#^*p* < 0.01 by Student's *t-*test.

To elucidate the responsiveness of CCR5^+^CD38^+^HLA-DR^+^CD8^+^ T cells toward glioma, CD8^+^ T cells were isolated from HD or patient's PBMCs and then co-cultured with the U87 glioma cell line for 24 h. CD8^+^ T cells in the supernatant were subjected to flow cytometry analysis, whereas U87 gliomas attached to the bottom of the wells were subjected to western blot analysis. Results of the flow cytometry analysis revealed an increase of TEMRA^+^ (CD45RA^+^CCR7^−^) and T-bet^+^ distributions in the CCR5^+^CD38^+^HLA-DR^+^CD8^+^ T-cell subset after co-culture (Figures 6A–C). A significant increase in PD-1 expression was also observed in CCR5^+^CD38^+^HLA-DR^+^CD8^+^ T cells prior to and after co-culture, whereas Tim-3 expression in CCR5^+^CD38^+^HLA-DR^+^CD8^+^ T cells was not significantly increased after co-culture (Figure 6C). Several effector molecules, including CD107a, IFN-γ, and Granzyme B, coincided significantly with CCR5^+^CD38^+^HLA-DR^+^CD8^+^ T cells after contact with the U87 glioma cells (Figures 6D,E). Additionally, results of flow cytometry analysis showed that CCR5^+^CD38^+^HLA-DR^+^CD8^+^ T cells of HD were unresponsive toward the U87 cells, which ruled out the possibility of allograft rejection (Figures 6B,C,E).

## Discussion

Suppression of adaptive immune activation can occur both systemically and locally in gliomas and other cancers such as melanoma (1, 31). Analysis from our patient group indicated that the reduced percentage of T cells in the peripheral blood was prominent and had a clear relation with disease severity. This phenomenon represents systemic immune modulation occurring in glioma. On the other hand, the presence of tumor-infiltrating effector CD8^+^ T cells was determined in prior studies in human glioma and mouse models to be involved in glioma eradication and to prompt certain anti-tumoral immune responses (8, 9, 32). The higher percentage of CD8^+^ T cells and the ratio to CD4^+^ T cells in TILs have been reported to be associated with a favorable prognosis in patients with glioma (11). However, the role of effector CD8 T cells in glioma is still obscure. Understanding whether a subset of effector CD8 T cells will infiltrate into tumors, their activity, and how they are suppressed might help identify immune-related therapeutic targets for intervention. The current study revealed that a subset of effector CD8^+^ T cells characterized as CD38^+^HLA-DR^+^CD8^+^ existed in both the peripheral blood and the tumor microenvironment, which indicated that the immune system of patients with glioma could recognize the tumor cells at an early stage. The high-grade gliomas appeared to generate a higher percentage of these cells, and these cells demonstrated increased infiltration into the tumors. Our data showed that the chemokine CCL5-CCR5 axis may be an important mechanism for attracting these effector T cells to the tumor microenvironment since tumor tissues expressed abundant CCL5 and T cells highly expressed CCR5. We also showed that CD38^+^HLA-DR^+^CD8^+^ cells were still functional, as they could be activated *ex vivo*. In response to glioma, CCR5^+^CD38^+^HLA-DR^+^CD8^+^ T cells were activated with a coinciding increase in TEMRA^+^ (CD45RA^+^CCR7^−^). Increases of IFN-γ and Granzyme B underlying CCR5^+^CD38^+^HLA-DR^+^CD8^+^ T cell activation were observed after co-culturing with glioma cells. In addition, the CD38^+^HLA-DR^+^CD8^+^ cells expressed higher levels of PD-1, and the presence of CD8^+^ T cells indicated reinforcement of glioma PD-L1 expression, suggesting that the PD-1/PD-L1 loop may facilitate the maintenance of proper immune responses. As such, therapies that can modulate the immune check-point may rescue these cells and would have potential benefits in the treatment of glioma.

The percentage of CD38^+^HLA-DR^+^CD8^+^ T-cell activation was significantly elevated in the circulating blood and tumors, indicating the active infiltration of these cells into the tumors. In addition, the elevated expression of CCL5 was also observed in patients with GBM, and this may be associated with an increased expression of CCR5 on CD38^+^HLA-DR^+^CD8^+^ T cells. This is consistent with previous studies that have reported that the expression of CCR5 facilitates CD8^+^ T-cell migration to damaged tissues (23, 33). The expression of CCR5 on CD8^+^ T cells was determined to promote the M1 macrophage and T-helper cell immune response associated with β chemokines, including CCL5 (34, 35). Although previous studies have demonstrated that CCR5 expression is restricted to memory (CD28^+^CD45RA^−^) and effector (CD28^−^CD45RA^−^ and CD28^−^CD45RA^+^) CD8^+^ T cells (23), in this study we also demonstrated that CCR5 was distinctly expressed in the CD38^+^HLA-DR^+^CD8^+^ T-cell population characterized as TEMRA^+^ (CD45RA^+^CCR7^−^) from patients with glioma. In addition, expression of TNFR2^+^ in the CD38^+^HLA-DR^+^CD8^+^ T-cell population appeared to be tumor specific, as these cells were not significantly potentiated in the patient PBMCs. In addition, the regulation of CCR5 in human peripheral blood lymphocytes has been shown to be inversely regulated by TNFα via TNFR2 activation (33). Results have also revealed the tumor-specific activation of TNFR2 in CD38^+^HLA-DR^+^CD8^+^ TILs. The regulation between CCR5 and TNFR2 appears to be consistent with past results as being inversely expressed in CD38^+^HLA-DR^+^CD8^+^ T cells from patient PBMCs (Figures 3A,B). However, this reduction of TNFR2^+^ on CD38^+^HLA-DR^+^CD8^+^ T cells from patient PBMCs was not equally observed in CD38^+^HLA-DR^+^CD8^+^ TILs, which displayed a marked increase in TNFR2^+^ expression (Figures 3A,B). The activation of TNFR2 was previously indicated to initiate cell survival via the NF-κB pathway, which leads to the induction of anti-apoptotic factors such as BCL-2 (36). Moreover, TNFR2-deficient mice reveal significant tumor growth, and the presence of TNFR2 associates with the accumulation of CD8^+^ TILs and CD8^+^ T-cell IFN-γ synthesis, suggesting TNFR2 potentiation on CD8^+^ TILs has a role in anti-tumor activities (37, 38).

Accumulating evidence has indicated that the CCL5-CCR5 interaction facilitates cancer progression, including breast cancer, osteosarcoma, and colon cancer (39). Previously, CCL5 expression in human and murine colon cancers was demonstrated to enhance regulatory T cell (Treg) infiltration through CCR5 expression, which can initiate TGF-β-dependent CD8^+^ T-cell apoptosis (40). In the present study, we also found an elevation of CCL5 in the glioma microenvironment, which is in accordance with the infiltration of CD38^+^HLA-DR^+^CD8^+^ TILs via CCR5 expression. However, it is unclear whether CCL5 in gliomas would also trigger similar Treg inhibition toward CD8^+^ TILs, as we only found a negligible population (<2%) of Treg characterized as Foxp3^+^ CD4^+^ (data not shown). Several reports have also indicated that the expression of immune checkpoints, including Tim-3 and PD-1, render CD8^+^ T cells exhausted, and PD-1 blockade can not only improve CD8^+^ TIL activation and anti-tumor efficacy (41) but can also result in a CD8^+^ T-cell-dependent survival benefit (42). Here, we have observed an increased expression of PD-1 on CD38^+^HLA-DR^+^CD8^+^ cells relative to the rest of the CD8^+^ cells, suggesting the possibility of CD38^+^HLA-DR^+^CD8^+^ TIL exhaustion. Furthermore, the expression of CCR5 has been indicated as a co-factor for aiding virus entry (e.g., HIV and cytomegalovirus) into immune cells, and this can interrupt systematic immune coherence (43, 44). However, whether or not elevated CCR5 expression in patients with glioma enforces susceptibility toward virus infection still requires further investigation.

The current study also revealed increased activation of effector CD38^+^HLA-DR^+^CD8^+^ T cells accompanied by sustained IFN-γ and IL-2 production, and these CD8^+^ T-cell subsets were functionally capable after co-stimulation with PMA/ION. Accumulating evidence has indicated that cytokines, including IFN-γ and IL-12, are essential for maximal effector CD8^+^ T-cell accumulation and for sustaining effector differentiation (45, 46). It was shown that exposure of CD8^+^ T cells to IL-12 and IFN-γ maintains the surface expression of the IL-2 receptor, which prolongs the CD8^+^ T-cell response to IL-2 and allows for sustained division of active CD8^+^ T cells, resulting in greater expansion of antigen-specific cells (46). The peripheral cytotoxic CD8^+^ T cells were shown to propagate a sustained basal level of IFN-γ and IL-2 under disease states with heterogeneity effects in diseases and treatment results (47–49). In addition, IL-2 is thought to prompt effector T-cell differentiation (13). Our current results revealed that the basal levels of IFN-γ and IL-2 were higher in the patient group, which may explain the hyperactivation of effector CD38^+^HLA-DR^+^CD8^+^ T cells in these patients. The exact role of sustained basal production of IFN-γ and IL-12 and its effect on antigen-specific cell expansion in patients with glioma remains unclear, but it may allow for persistent pathogen control. Our result also revealed that the lymphopenic state was notable in these patients. The relationship between lymphopenia and the burst activation of CD38^+^HLA-DR^+^CD8^+^ T cells in high-grade glioma remains uncertain. Several reports have demonstrated that naïve T lymphocytes undergo rapid expansion during the lymphopenic state, requiring the engagement of MHCs and T cell receptors (TCR) (50, 51). The hyperexpansion of CD8^+^ T cells driven by lymphopenia may transiently display effector functions prior to differentiation of memory-like T cells, which is coupled to the presence of CD44, CD122 (interleukin 2 receptor β), and Ly6C to secret IFN-γ (52, 53).

In this present study, we demonstrated the functional expression of CCR5 on CD38^+^HLA-DR^+^CD8^+^ T cells and the presence of CD45RA^+^CCR7^−^ underling CCR5^+^CD38^+^HLA-DR^+^CD8^+^ T-cell activation in patients with glioma. The elevated TNFR2^+^CD38^+^HLA-DR^+^CD8^+^ TILs indicated that TNFR2 might be involved in the anti-tumor response. Detection of CCL5 in GBM TILs suggested CCL5 might be one of the β chemokines that attract CD38^+^HLA-DR^+^CD8^+^ TIL penetration. The PD-L1 expression level in GBM glioma appears to be preserved by the presence of CD8^+^ T cells, which presumably limits the effectiveness of CCR5^+^CD38^+^HLA-DR^+^CD8^+^ TILs; therefore, the availability of CCR5^+^CD38^+^HLA-DR^+^CD8^+^ T cells in term of numbers to initiate proper immune responses may also be violated and insufficient. As crucial central nervous system antigen-presenting cells, the effect of microglia on the propagation of CD38^+^HLA-DR^+^CD8^+^ T cells may be a key factor that delineates the antigen-specific T-cell niche. Further studies of CCR5 on microglia will help to clarify the effect of CD38^+^HLA-DR^+^CD8^+^ T cells on the anti-tumor response.

## Data Availability Statement

The raw data supporting the conclusions of this manuscript will be made available by the authors, without undue reservation, to any qualified researcher.

## Ethics Statement

This study was approved by the Chang Gung Medical Foundation Institutional Review Board (No. 102-1096B) and in accordance with the Helsinki Declaration. Patients were recruited from the Department of Neurosurgery, Chang Gung Medical Foundation, Taiwan, between 2013 and 2016. All eligible patients were informed about the details of this study and provided a signed informed consent prior to study participation.

## Author Contributions

CW, C-YL, J-YF, and P-YC were involved in the conception and design of the study. CW, H-CC, and L-YF performed the experiments. C-YL and J-HF provided technical support. CW, L-YF, C-YL, C-YH, and P-YC analyzed the data. K-CW and P-YC provided important materials. C-YL and J-YF critically revised the manuscript. CW and P-YC wrote the manuscript.

### Conflict of Interest

The authors declare that the research was conducted in the absence of any commercial or financial relationships that could be construed as a potential conflict of interest.
