# Supplementary material for: Functional Change of Effector Tumor-Infiltrating CCR5+CD38+HLA-DR+CD8+ T Cells in Glioma Microenvironment
Source: Front Immunol. 2019 Oct 9;10:2395. doi: 10.3389/fimmu.2019.02395 (PMC6794477; doi:10.3389/fimmu.2019.02395)
Supplement: Supplementary file 1 [file Data_Sheet_1.docx]

Supplementary Material

**Supplementary Table 1.** Descriptive statistics of the study population and mean age differences.

| **Group Statistics** | | | | | |
| --- | --- | --- | --- | --- | --- |
|  | G2 | N | Mean | Std. Deviation | Std. Error Mean |
|  | Healthy donor | 36 | 46.8889 | 15.51364 | 2.58561 |
|  | Patient | 143 | 51.8741 | 13.73382 | 1.14848 |

**Supplementary Table 2.** Results of Independent *t* test for age differences between healthy donor and patient groups.

| **Independent Samples Test** | | | | | | | | | |
| --- | --- | --- | --- | --- | --- | --- | --- | --- | --- |
|  | Levene's Test for Equality of Variances | | t-test for Equality of Means | | | | | | |
|  |  | |  | | | | | 95% Confidence Interval of the Difference | |
|  | F | Sig. | t | df | Sig. (2-tailed) | Mean Difference | Std. Error Difference | Lower | Upper |
| Equal variances assumed | 1.697 | .194 | -1.876 | 177 | .062 | -4.92968 | 2.62781 | -10.11554 | .25618 |
| Equal variances not assumed |  |  | -1.747 | 49.799 | .087 | -4.92968 | 2.82138 | -10.59716 | .73779 |

**
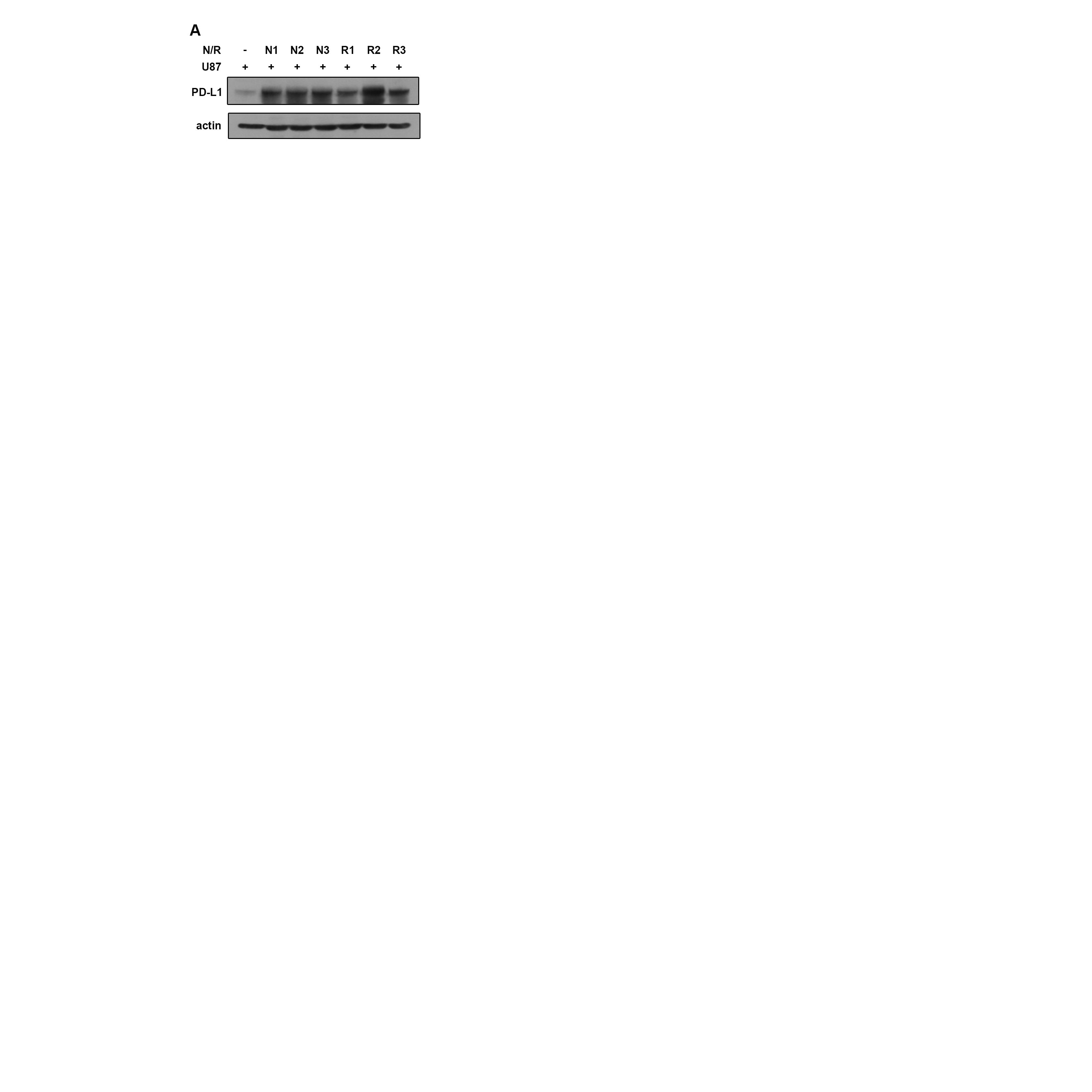
**

**Supplementary Figure 1.** CD8^+^ T cells of newly-diagnosed (n = 3) and recurrent (n = 3) patients enforce glioma PD-L1 expression. N = newly-diagnosed; R = recurrent.
